# An explainable ensemble machine learning framework for predicting cartilage biomechanical degradation from transcriptomic profiles: toward smart orthopedic device design

**DOI:** 10.3389/fbioe.2026.1831264

**Published:** 2026-05-01

**Authors:** Yelda Fırat

**Affiliations:** Department of Computer Engineering, Mudanya University, Bursa, Mudanya, Türkiye

**Keywords:** cartilage degradation index, machine learning, mechanotransduction, osteoarthritis, SHAP analysis, smart orthopedic implants

## Abstract

**Introduction:**

Osteoarthritis (OA) is a common joint disease characterized by progressive biomechanical deterioration of cartilage tissue. This study aims to develop an explainable ensemble machine learning model capable of predicting cartilage biomechanical deterioration from transcriptomic profiles and to present this model as a digital biomarker for intelligent orthopedic devices.

**Methods:**

Seven independent datasets (n = 203) obtained from the Gene Expression Omnibus (GEO) database were harmonized using the Joint ComBat algorithm. Differentially expressed gene (DEG) analysis performed on the training cohort (n = 122) identified 93 significant genes. An ensemble model combining Random Forest (RF), XGBoost, and Support Vector Machine (SVM) algorithms was trained using these genes. The disease probability generated by the model was redefined as a continuous Cartilage Degradation Index (CDI). The model’s performance and generalizability were tested on four independent validation cohorts including patients undergoing arthroscopic partial meniscectomy (APM, n = 44), chondrocytes subjected to *in vitro* mechanical stress (n = 18), and different geographic cohorts (n = 19). The biological basis of the model’s decisions was elucidated using SHAP (SHapley Additive exPlanations) analysis and comprehensive pathway enrichment analyses.

**Results:**

The ensemble model demonstrated a 5-fold cross-validation performance with an area under the curve (AUC) of 0.960. Permutation testing (p < 0.001) and bootstrap 95% confidence interval (CI) (AUC: 0.900–0.996) confirmed the model’s robustness. CDI modeled degradation across a biologically consistent spectrum: 0.03–0.09 in healthy cartilage, 0.30–0.42 in early-stage APM, and 0.79 in advanced-stage OA. CDI scores were significantly higher in cells exposed to abnormal mechanical stress (overstress) compared to the control group (0.60 vs. 0.52, AUC = 0.778). SHAP analysis highlighted COL15A1, CXCL14, CISH, FAP, and PIM2 as the most critical mechanosensitive genes. Pathway analysis confirmed that the model is based on mechanotransduction mechanisms such as focal adhesion, extracellular matrix (ECM) organization, and epithelial–mesenchymal transition.

**Conclusion:**

This study presents a transcriptomic CDI capable of measuring cartilage biomechanical degradation at the molecular level. The identified mechanosensitive gene profile holds strong potential as a digital biomarker for next-generation smart orthopedic implants and wearable biomechanical sensors.

## 1 Introduction

Osteoarthritis (OA) is the most common chronic joint disease worldwide, characterized by progressive destruction of articular cartilage, subchondral bone changes, and synovial inflammation (Loeser et al., 2012; Martel-Pelletier et al., 2016). According to the Global Burden of Disease studies, the prevalence of OA is rapidly increasing due to the aging population and rising obesity rates, and it is projected to affect approximately one billion people worldwide by 2050 (Safiri et al., 2020; GBD and Osteoarthritis Collaborators, 2023). While the pathogenesis of the disease has traditionally been viewed as a *wear and tear* process, current literature reveals that OA is a complex biomechanical and biochemical disorder involving all joint tissues (Loeser et al., 2012). In particular, disruption of the biomechanical integrity of articular cartilage is a critical trigger that occurs in the early stages of the disease and leads to irreversible structural damage (Sanchez-Adams et al., 2014).

Chondrocytes, the basic building blocks of cartilage tissue, are *mechanosensitive* cells that sense mechanical loads on the joint and maintain a balance between extracellular matrix (ECM) synthesis and degradation (O’Conor et al., 2014; Wang et al., 2023). Under healthy physiological load conditions, chondrocytes produce anabolic signals to maintain cartilage homeostasis, while abnormal mechanical stress (e.g., trauma, obesity, or joint instability) activates catabolic pathways, leading to ECM degradation (Zhao et al., 2020). This mechanotransduction process involves the conversion of mechanical signals into gene expression changes via focal adhesion complexes and ion channels (Shin et al., 2016). In the presence of inflammatory mediators, structural changes in the ECM are further accelerated, significantly reducing the load-bearing capacity of the cartilage (Maldonado and Nam, 2013). Therefore, understanding the transcriptomic signatures associated with biomechanical degradation of cartilage tissue is of great importance for early diagnosis and for developing targeted therapeutic strategies. It is important to note that in this study, *biomechanical degradation* refers to the molecular and cellular responses to abnormal mechanical stress, rather than direct physical measurements of tissue mechanics.

In recent years, the development of high-throughput transcriptomic technologies has made significant contributions to elucidating OA pathogenesis at the molecular level. Publicly available databases such as the Gene Expression Omnibus (GEO) provide extensive gene expression profiles of healthy and osteoarthritic cartilage tissues (Ramos et al., 2014; Dunn et al., 2016). Furthermore, transcriptomic responses of chondrocytes to biomechanical loads have begun to be characterized in detail using *in vitro* mechanical stress models (Hung et al., 2021). However, the analysis of these large datasets is often limited by traditional differentially expressed gene (DEG) methods, and the *batch effect* problem between data obtained from different platforms (microarray, RNA-seq) restricts the generalizability of the results (Bolstad et al., 2003; Johnson et al., 2007). In this context, there is a growing need for machine learning (ML) approaches that can integrate multiple cohort data and model complex genetic interactions (Zhao et al., 2024).

Machine learning algorithms, particularly Random Forest (RF) (Breiman, 2001), XGBoost (Chen and Guestrin, 2016), and Support Vector Machines (SVM) (Cortes and Vapnik, 1995), demonstrate superior performance in discovering hidden patterns in high-dimensional omics data (Zhao et al., 2024). However, the *black-box* nature of ML models in medical applications is one of the biggest obstacles to their clinical acceptability. Developed to overcome this problem, the SHAP (SHapley Additive exPlanations) method, based on game theory, can explain the contribution of each feature (gene) to model predictions at the individual level, thereby increasing the transparency of the models (Lundberg and Lee, 2017; Lundberg et al., 2018; Lundberg et al., 2020). Although the integration of ML and transcriptomic data in the field of OA offers promising diagnostic biomarkers (Mobasheri, 2016), current studies generally focus only on disease classification and do not evaluate gene expression profiles as a continuous *biomechanical degradation spectrum*.

The primary objective of this study is to develop an explainable ensemble machine learning model capable of detecting biomechanical degradation at an early stage using transcriptomic profiles of cartilage tissue. To this end, seven independent datasets from the GEO database were harmonized, and the disease probability score generated by the model was conceptualized as a continuous *Cartilage Degradation Index* (CDI). The performance of the developed model was validated in independent validation cohorts such as patients undergoing arthroscopic partial meniscectomy (APM) and chondrocytes subjected to *in vitro* mechanical stress. Furthermore, the model’s decisions were biologically interpreted and novel mechanosensitive genes were identified using SHAP analysis and comprehensive pathway enrichment analyses (Chen et al., 2013; Kuleshov et al., 2016; The Gene Ontology Consortium, 2019; Kanehisa et al., 2021). The findings indicate that the developed CDI score and the identified mechanosensitive gene profile provide a foundational computational framework for the future design of next-generation smart orthopedic implants. While direct *in vivo* transcriptomic monitoring remains technologically challenging, translating these molecular signatures into detectable protein or metabolite biomarkers in synovial fluid could eventually enable smart implants equipped with biochemical sensors to dynamically monitor joint health.

The remainder of this manuscript is organized as follows: Section 2 details the data collection, preprocessing, model development, and validation strategy; Section 3 presents the model performance, CDI validation, SHAP analysis, and pathway enrichment results; Section 4 discusses the findings within the context of the current literature; and Section 5 concludes the study.

## 2 Methods

### 2.1 Study design and data acquisition

In this study, a total of seven independent datasets (n = 203) from the GEO database were integrated to predict the molecular signatures of cartilage biomechanical degradation from gene expression profiles. All datasets were obtained from human knee articular cartilage and chondrocytes, representing biomechanical degradation caused by osteoarthritis (OA) and mechanical stress. To improve the generalizability of the model and prevent overfitting, the data were divided into five different cohorts (Table 1): model training, internal validation, external testing, mechanotransduction validation, and additional stability testing.

**TABLE 1:** Summary of datasets used in the study.

Cohort role	GEO accession	Platform	Tissue type	Sample size	Groups (diseased vs. healthy)
Training	GSE57218	Microarray	Knee articular cartilage	73	OA (33) vs. preserved (33) + healthy (7)
Training	GSE114007	RNA-seq	Knee cartilage	38	OA (20) vs. normal (18)
Training	GSE169077	Microarray	Knee cartilage	11	Advanced OA (6) vs. normal (5)
Internal validation	GSE117999	Microarray	Knee cartilage	20	OA (10) vs. APM/Early-stage (10)
External test	GSE98918	Microarray	Knee cartilage	24	OA (12) vs. APM/Early-stage (12)
Mechanotransduction	GSE165874	RNA-seq	iPSC-chondrocyte	18	Mechanical stress (9) vs. control (9)
Stability test	GSE129147	Microarray	Knee cartilage	19	Damaged (10) vs. intact (9)
Total	​	​	​	203	Diseased (100) vs. healthy/Control (103)

APM, arthroscopic partial meniscectomy.

A large and heterogeneous training cohort of 122 samples was created by combining three different datasets (GSE57218, GSE114007, GSE169077) to train the machine learning model. A binary classification strategy was adopted in this cohort, dividing samples into two groups: *OA (biomechanically impaired, n = 59)* and *Non-OA (healthy or preserved, n = 63)*. Specifically, the inclusion of *preserved* cartilage samples from areas of the joints of OA patients that had not yet shown macroscopic damage into the Non-OA group was strategically chosen to prevent the model from overfitting to inter-patient genetic variations and to allow it to directly learn the actual biomechanical and molecular signals specific to cartilage degradation.

The generalizability and biological validity of the model were tested in four independent validation cohorts that had never been used in training. Cohorts GSE117999 (n = 20) and GSE98918 (n = 24) were used for internal validation and external testing, respectively. To verify whether the genes identified by the model responded to mechanical stress, cohort GSE165874 (n = 18), containing cartilage cells exposed to cyclic tensile strain *in vitro*, was selected for mechanotransduction validation. Finally, further stability validation was performed by testing the model’s performance on cohort GSE129147 (n = 19), an independent cohort from Turkey (Aşık et al., 2020). Details of all datasets used in the study and cohort distributions are summarized in Table 1.

### 2.2 Data preprocessing and batch effect correction

Since the seven different datasets included in the study were generated by independent research groups using different technologies (Illumina, Affymetrix, Agilent microarray platforms, and RNA-seq), an extensive preprocessing pipeline was applied before data integration. First, the different gene nomenclature systems (Probe ID, Ensembl ID, etc.) in each dataset were converted to the universal Gene Symbol (HUGO) format using up-to-date annotation files obtained from the NCBI and Ensembl BioMart databases. If more than one probe corresponded to the same gene symbol, the expression values ​​of these probes were averaged. Then, to ensure that the machine learning model could run in all cohorts, the intersection of the datasets was taken, and 10,283 genes common to all seven datasets were identified, and all matrices were restricted to this gene set.

To equalize the statistical distributions of data from different platforms and minimize the effect of outliers, each dataset was subjected to quantile normalization individually. However, technical variance (batch effect) resulting from the production of datasets at different times and in different laboratories was evaluated using Principal Component Analysis (PCA), and it was observed that the first principal component (PC1) explained 82% of the variance, and that the samples clustered according to the dataset they originated from, rather than according to disease status. To eliminate variance and reveal the biological signal, the parametric ComBat (Empirical Bayes) algorithm was applied, where each dataset was defined as a separate *batch* (Johnson et al., 2007). PCA analysis after ComBat correction confirmed that dataset-specific clustering was completely eliminated, PC1 variance dropped to 17.2%, and samples were homogeneously distributed regardless of dataset. Finally, the three datasets selected for model training (GSE57218, GSE114007, GSE169077) were combined into a single matrix to create the final training set consisting of 10,283 genes and 122 samples.

### 2.3 DEG analysis

Differential gene expression analysis is a fundamental bioinformatics approach used to identify genes that show statistically significant differences in expression between two or more biological conditions (Ramos et al., 2014). In this study, an independent samples Welch’s *t*-test was applied to determine DEGs between OA (n = 59) and Non-OA (n = 63) groups in the training cohort (n = 122) harmonized with ComBat. The raw p-values calculated for each gene were corrected for False Discovery Rate (FDR) using the Benjamini–Hochberg method as part of multiple testing correction (Benjamini and Hochberg, 1995). For a gene to be considered differentially expressed, two thresholds commonly accepted in transcriptomic studies had to be met simultaneously (Zhao et al., 2024; Dunn et al., 2016). These were: corrected p-value (FDR) < 0.05 and absolute log2 fold change (|log2FC|) > 0.5. The FDR <0.05 threshold ensures statistical reliability by controlling the false positive rate, while the |log2FC| > 0.5 threshold selects expression differences that are significant not only statistically but also biologically. According to these criteria, 93 genes were identified as differentially expressed out of 10,283 genes; 74 of these were classified as upregulated (increased expression) and 19 as downregulated (decreased expression) in the OA group. These 93 identified DEGs were then used as the feature set for the machine learning model in subsequent stages.

### 2.4 Machine learning model development

**FIGURE 1:**
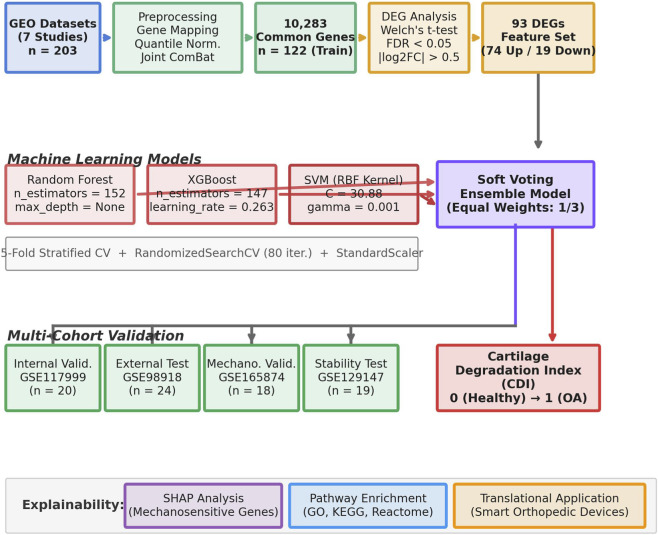
Model architecture.

To predict biomechanical degradation in cartilage tissue, an ensemble architecture was established using 93 selected DEGs, combining RF, XGBoost, and SVM algorithms with a soft voting strategy. The overall flowchart and data processing pipeline of the developed model are summarized in Figure 1.

As shown in Figure 1, the developed framework consists of four main stages: data preprocessing, feature selection and model training, multi-cohort validation, and biological explainability.

#### 2.4.1 Feature selection and algorithm choice

The model’s feature set consists of 93 DEGs that were identified in previous stages and proven to have biological significance. This dimensionality reduction strategy was deliberately chosen to avoid the *curse of dimensionality* that arises in high-dimensional data structures and to reduce the risk of model overfitting. For the classification task, three different machine learning algorithms that have proven successful in transcriptomic data analysis were selected: Random Forest (RF), XGBoost, and Support Vector Machine (SVM). The rationale for selecting these algorithms is that they represent three fundamentally different learning paradigms: bagging (RF), gradient boosting (XGBoost), and margin maximisation (SVM). By combining these diverse approaches, the ensemble model minimises the inductive bias inherent to any single algorithm, captures both linear and complex non-linear gene interactions, and produces more stable predictions across heterogeneous datasets.

#### 2.4.2 Hyperparameter optimization and ensemble strategy

To ensure the reliability and generalizability of the model, a 5-fold stratified cross-validation method was used in the training process. A stratified approach was specifically chosen to preserve the original class distribution (OA vs. Non-OA) across all folds, which is critical for maintaining stable performance estimates in a relatively modest sample size. During each fold, hyperparameter tuning was performed exclusively on the training split using RandomizedSearchCV (80 iterations). This randomised search was preferred over an exhaustive grid search to efficiently explore a wider hyperparameter space while mitigating the risk of overfitting. Furthermore, to prevent data leakage, feature scaling (StandardScaler) was strictly applied within the cross-validation loop, ensuring that the validation fold remained completely unseen during the scaling process. For RF, the ranges were scanned as follows: n_estimators [100–500], max_depth [3, 5, 7, 10, None], and max_features [sqrt, log2, 0.3–0.7]; for XGBoost, the ranges were scanned as follows: n_estimators [50–400], max_depth [2–8], learning_rate [0.01–0.31], and subsample [0.5–1.0]; for SVM, the ranges were scanned as follows: C [0.01–50], gamma [10^−4^–10^−1^], and kernel [rbf, linear]. The best hyperparameters obtained as a result of optimization are summarized in Table 2.

**TABLE 2:** Hyperparameter optimization results (RandomizedSearchCV, 80 iterations).

Model	Best CV AUC	n_estimators	max_depth	Key parameters	Time (s)
Random forest	0.9554	152	None	max_features = sqrt, min_samples_leaf = 1	56.9
XGBoost	0.9514	147	6	lr = 0.263, subsample = 0.53, gamma = 0.970	3.6
SVM	0.9833	—	—	C = 30.88, kernel = rbf, gamma = 0.001	0.9

As shown in Table 2, the SVM algorithm achieved the highest cross-validation performance among the individual models (AUC = 0.9833). Random Forest reached an AUC of 0.9554 with 152 trees and no depth limitation (max_depth = None), while XGBoost showed an AUC of 0.9514 with 147 trees and six levels of depth. To prevent data leakage during the optimization process, feature scaling (StandardScaler) was applied independently within each cross-validation layer (fold). To balance the weaknesses of the individual models and increase prediction stability, the optimized RF, XGBoost, and SVM models were combined using a *soft voting* strategy to create the final ensemble model. In this strategy, the final prediction was obtained by taking the arithmetic mean (equal weighting: 1/3) of the class probabilities produced by each base model. The model’s performance was evaluated using Area Under the Receiver Operating Characteristic Curve (AUC–ROC), F1-score, sensitivity, and specificity metrics.

### 2.5 Validation strategy and cartilage degradation index

To evaluate the generalizability and biological validity of the developed machine learning model, a comprehensive validation strategy was applied to four independent validation cohorts not included in the training process. Examination of the external validation cohorts (GSE98918 and GSE117999) revealed that the Control group in these datasets actually consisted of patients who had undergone APM. APM cartilage is not healthy tissue; it is tissue that has suffered early-stage mechanical damage and entered a degradation process. In line with this biological reality, instead of the traditional binary classification approach, a continuous metric reflecting the progressive nature of the disease was developed.

In this context, the disease probability score generated by the ensemble model has been redefined as the CDI. Specifically, the ensemble model takes the normalized expression values of the 93 selected DEGs as input and outputs a continuous probability score ranging from 0.0 to 1.0. This output is calculated using a soft-voting mechanism, which averages the predicted probabilities from the RF, XGBoost, and SVM base classifiers. Rather than applying a rigid binary threshold (e.g., >0.5 for OA), this continuous output is directly utilized as the CDI, allowing it to quantitatively reflect the gradual transition from healthy cartilage (values closer to 0) to advanced biomechanical degradation (values closer to 1). Within this new framework, APM samples have been reclassified as Early Stage Degradation instead of Healthy. The model’s success has been evaluated not only by its ability to distinguish between endpoints (completely healthy vs. advanced OA) but also by its capacity to accurately model the degradation spectrum (Healthy → APM → Advanced OA).

To validate the model’s sensitivity to mechanical stress, a mechanotransduction cohort (GSE165874) containing cartilage cells subjected to abnormal mechanical stress (overstress, 10% cyclic tensile strain) *in vitro* was used. Furthermore, to test the model’s stability under different geographical and technical conditions, an additional validation was performed on a stability cohort (GSE129147) obtained from Turkey and sequenced on a different platform (Affymetrix) (Aşık et al., 2020). This multiple-cohort validation strategy was designed to systematically test whether the CDI score accurately reflects different degradation stages, mechanical stress conditions, and geographical/technical heterogeneity.

### 2.6 SHAP and mechanosensitivity scoring

To make the decisions of the developed ensemble model biologically interpretable, the SHAP framework was used (Lundberg and Lee, 2017). SHAP is a model-agnostic explainable artificial intelligence method based on Shapley values derived from game theory, which calculates the marginal contribution of each feature to individual predictions (Lundberg et al., 2018). In this study, the computationally efficient TreeExplainer algorithm (Lundberg et al., 2020) was used for the RF and XGBoost models, and the KernelExplainer algorithm was used for the SVM model. The SHAP values of the ensemble model were obtained by taking the arithmetic mean of the SHAP matrices of the three base models.

Global feature significance was determined by calculating the mean absolute SHAP value (mean |SHAP|) of each gene across all samples. To validate the generalizability of the model, the consistency between SHAP-based gene significance rankings in the training cohort and four independent validation cohorts was assessed using Spearman rank correlation.

As one of the original contributions of the study, a Mechanosensitivity Score was developed to measure the sensitivity of each gene to mechanical stress. This score was calculated as the absolute value of the difference between the mean SHAP values of the mechanically stressed samples in the mechanotransduction cohort (GSE165874) and the control samples. The obtained scores were normalized to a range of 0–1, and the genes were divided into three categories: Highly Mechanosensitive (score >0.6), Moderately Mechanosensitive (0.3–0.6), and Low Mechanosensitive (<0.3). This classification was designed to quantitatively reveal the relationship of the genes that guide the model’s decisions with mechanical stress and to identify potential biomechanical biomarkers.

### 2.7 Pathway enrichment analysis

To systematically characterize the biological functions and associated signaling pathways of the 93 identified DEGs, a comprehensive pathway enrichment analysis was performed using the Enrichr web server (Chen et al., 2013; Kuleshov et al., 2016). Over-representation analysis (ORA) based on Fisher’s exact test was used for the enrichment analyses (Boyle et al., 2004). The analyses were conducted across six different ontologies and databases: three subcategories of the Gene Ontology (GO) database (Biological Process, Cellular Component, and Molecular Function) (The Gene Ontology Consortium, 2019), KEGG (Kanehisa et al., 2021), Reactome (Jassal et al., 2020), and MSigDB Hallmark gene sets (Liberzon et al., 2015). A statistical significance threshold of p-value (FDR) < 0.05 was applied (Benjamini and Hochberg, 1995). Furthermore, directional enrichment analyses were performed separately for upregulated (74 genes) and downregulated (19 genes) gene sets to distinguish between biological processes activated and repressed in OA.

As one of the original contributions of this study, a SHAP-weighted pathway score was developed in addition to the traditional p-value–based ranking. In this approach, the contribution of pathways to the prediction decisions of the machine learning model was quantitatively evaluated by averaging the SHAP-based feature importance values of the genes within each pathway. This method enabled the prioritization of biological mechanisms that actually drive the classification performance of the model among statistically significant pathways.

### 2.8 Software and statistical environment

All statistical analyses and machine learning models in this study were implemented using the Python 3.11 programming language. NumPy and Pandas were used for data preprocessing and manipulation; pyCombat was used for batch-effect correction; and the SciPy and statsmodels libraries were used for DEG analysis. Machine learning models were trained with the scikit-learn (v1.8) and XGBoost (v3.2) libraries, and the explainability of the models was ensured with the SHAP (v0.51) package. Pathway enrichment analyses were performed by connecting to the Enrichr API via GSEApy (v1.1). All visualizations were created with the Matplotlib and Seaborn libraries.

## 3 Results

In this section, the cross-validation performance of the developed ensemble machine learning model, CDI in independent validation cohorts, the SHAP-based gene importance analysis and mechanosensitivity scoring, and the pathway enrichment analysis results are presented, respectively.

### 3.1 Model performance and cross-validation results

**FIGURE 2:**
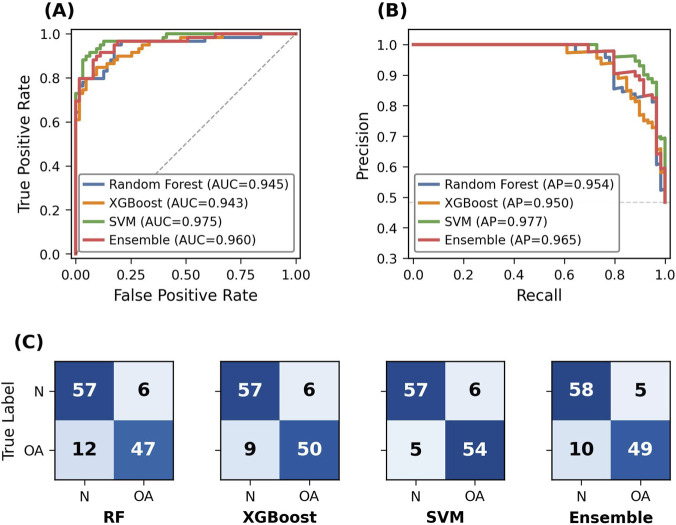
Cross-validation performance evaluation of individual and ensemble machine learning models for cartilage degradation classification. **(A)** ROC curves with AUC values for each model. **(B)** Precision-Recall curves with average precision (AP) scores. **(C)** Confusion matrices summarizing classification outcomes across all cross-validation folds.

The performance of machine learning models developed to predict biomechanical degradation in cartilage tissue was evaluated using a 5-fold stratified cross-validation method. In this framework, the classification capabilities of RF, XGBoost, and SVM, as well as an ensemble model combining these algorithms using a *soft voting* strategy, were analyzed comparatively. Model performance was evaluated using the AUC–ROC, Precision–Recall Curve, F1-score, and accuracy metrics, and the results are visualized in Figure 2.

As shown in Figure 2, all developed models exhibited high discrimination performance. Examining the ROC curves presented in Figure 2A, SVM (AUC = 0.975) showed the highest performance among the individual models, followed by Random Forest (AUC = 0.945) and XGBoost (AUC = 0.943). The ensemble model, which combined the prediction probabilities of the three models, showed a very strong and balanced performance with an AUC of 0.960. The Precision–Recall curves in Figure 2B confirm the models’ robustness against possible class imbalances in the dataset; here, SVM (AP = 0.977) and the ensemble model (AP = 0.965) achieved the highest average precision values. The confusion matrices in Figure 2C show the distribution of classification errors for the models. The SVM model achieved the highest sensitivity by correctly classifying 54 out of 59 positive (OA) cases, while the ensemble model achieved the highest specificity by correctly classifying 58 out of 63 negative (Healthy/Normal) cases. Cross-validation performance metrics for the models are summarized in detail in Table 3.

**TABLE 3:** Cross-validation performance metrics (5-Fold stratified CV).

Model	Accuracy	Precision	Recall	F1	AUC	Brier score	Train AUC	CV-train gap
Random forest	0.8525	0.8868	0.7966	0.8393	0.9454	0.0948	1.0000	0.0546
XGBoost	0.8770	0.8929	0.8475	0.8696	0.9427	0.0963	1.0000	0.0573
SVM	0.9098	0.9000	0.9153	0.9076	0.9750	0.0645	1.0000	0.0250
Ensemble (RF + XGBoost + SVM)	0.8770	0.9074	0.8305	0.8673	0.9596	0.0780	1.0000	0.0404

As shown in Table 3, the SVM model stands out among the individual models with 90.98% accuracy and an F1 score of 0.9076. The ensemble model achieved 87.70% accuracy and an F1 score of 0.8673. When the AUC difference (CV–Train Gap) between the training and validation sets is examined, this difference is only 0.0250 for SVM and 0.0404 for the ensemble model. These low differences indicate that the models do not overfit the training data and have high generalizability. In addition, in terms of the Brier score, which measures the calibration of the predictions, SVM (0.0645) and the ensemble model (0.0780) have the lowest (best) error rates.

To provide additional evidence of model robustness given the modest sample size, a permutation test (1,000 iterations) was conducted. The results of this analysis are presented in Table 4.

**TABLE 4:** Permutation test results (1,000 iterations) for model robustness.

Model	True AUC	Mean permuted AUC	p-value
Random forest	0.953	0.496	<0.001
XGBoost	0.937	0.496	<0.001
SVM	0.983	0.493	<0.001
Ensemble	0.963	0.496	<0.001

As seen in Table 4, the permutation test confirmed that all models captured true biological signals rather than chance artifacts. The true AUC of the ensemble model (0.963) was significantly higher than the mean permuted AUC (0.496), yielding a highly significant p-value (p < 0.001). This result demonstrates that the model’s predictive power is not attributable to random patterns in the data.

Furthermore, to evaluate the stability of the model’s predictive performance, bootstrap confidence interval (CI) estimation (1,000 iterations) was performed. The results are presented in Table 5.

**TABLE 5:** Bootstrap 95% CIs (1,000 iterations).

Model	AUC mean	AUC 95% CI	F1 mean	F1 95% CI
Random forest	0.947	0.888–0.990	0.851	0.750–0.944
XGBoost	0.933	0.863–0.984	0.844	0.732–0.933
SVM	0.968	0.917–0.998	0.881	0.781–0.957
Ensemble	0.958	0.900–0.996	0.870	0.762–0.957

CI, confidence interval.

As seen in Table 5, the bootstrap analysis demonstrated narrow 95% CIs for the ensemble model (AUC: 0.900–0.996), indicating high stability and robustness despite the relatively modest sample size. The consistently high lower bounds across all metrics confirm that the model maintains reliable predictive performance.

The results of the ablation study performed to validate the architecture of the ensemble model are presented in Table 6.

**TABLE 6:** Ablation study: model configuration comparison.

Model Configuration	AUC	F1	Accuracy
RF only	0.9454	0.8393	0.8525
XGBoost only	0.9427	0.8696	0.8770
SVM only	0.9750	0.9076	0.9098
RF + XGBoost	0.9440	0.8850	0.8934
RF + SVM	0.9656	0.9060	0.9098
XGBoost + SVM	0.9634	0.8870	0.8934
RF + XGBoost + SVM (ensemble)	0.9596	0.8673	0.8770

As shown in Table 6, in this analysis comparing single, double, and triple model combinations, SVM alone (AUC = 0.975) or in combination with RF (AUC = 0.965) produced marginally higher AUC values. However, the main reason for preferring the triple ensemble model (RF + XGBoost + SVM) is that it balances the weaknesses of a single algorithm by combining different algorithmic approaches (decision trees, gradient boosting, and margin maximization) and produces more stable predictions in independent validation cohorts. The ensemble model, with an AUC of 0.960, falls within the clinically strong performance band (>0.90), providing a reliable basis for detecting cartilage biomechanical deterioration.

### 3.2 CDI and external validation

**FIGURE 3:**
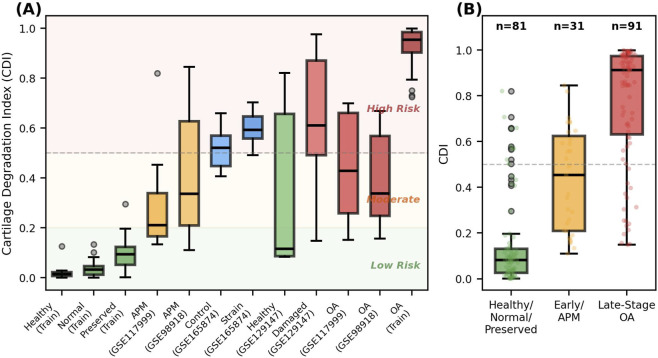
Cross-cohort distribution of the Cartilage Degradation Index (CDI). **(A)** CDI values across all conditions and cohorts with risk zone shading. **(B)** CDI distribution grouped by degradation stage.

Using the predictive probabilities of the developed ensemble model, CDI, which measures biomechanical deterioration in cartilage tissue on a continuous spectrum (0: Healthy, 1: Advanced-stage OA), was calculated. To test the generalizability of the model and the biological validity of the CDI score, a comprehensive validation study was conducted across four independent validation cohorts (GSE117999, GSE98918, GSE165874, GSE129147). These cohorts represent different stages of degradation (APM, OA), mechanical stress conditions (strain), and tissue stability (damaged). The distribution of the obtained CDI scores across cohorts and degradation stages is visualized in Figure 3.

As shown in Figure 3, the CDI score increases proportionally with the severity of cartilage tissue deterioration. Examining the cohort-based distributions presented in Figure 3A, CDI scores for healthy (Healthy/Normal/Preserved) samples in the training set are concentrated between 0.0 and 0.2 (low risk), while scores for advanced OA samples fall within the 0.8–1.0 (high risk) range. Partial meniscectomy (APM) patients in the external validation cohorts (GSE117999 and GSE98918) are located between the healthy and OA groups, showing a distribution in the intermediate-risk (0.2–0.5) region. In the analysis shown in Figure 3B, where all samples were grouped according to three main degradation stages, the mean CDI value for healthy tissues was 0.06, while this value increased to 0.36 in the APM group, representing early degradation, and to 0.79 in the advanced-stage OA group. This gradual increase supports the idea that the CDI score can be used as a continuous biomarker reflecting disease progression. Performance metrics in the independent validation cohorts are summarized in Table 7.

**TABLE 7:** External validation results (ensemble model).

Dataset	Accuracy	F1	AUC
Mechanotransduction (GSE165874) - control vs. strain	0.611	0.696	0.778
Stability (GSE129147) - damaged vs. healthy	0.684	0.700	0.767

As shown in Table 7, the ensemble model demonstrated particularly high discrimination performance in the mechanical stress (GSE165874) and tissue stability (GSE129147) cohorts. In the mechanotransduction cohort (Control vs. Strain), the model was able to distinguish tissues exposed to mechanical stress from healthy tissues with 61.1% accuracy and an AUC of 0.778. Similarly, in the stability cohort (Damaged vs. Healthy), an accuracy of 68.4% and an AUC of 0.767 were achieved. The mean CDI values and standard deviations calculated according to the conditions in all cohorts are detailed in Table 8.

**TABLE 8:** CDI summary by condition.

Dataset	Condition	Mean CDI	Std CDI	N
Training	Healthy	0.028	0.044	7
Training	Normal	0.037	0.035	23
Training	Preserved	0.094	0.063	33
Training	OA	0.931	0.070	59
Internal val (GSE117999)	APM	0.296	0.210	10
Internal val (GSE117999)	OA	0.443	0.217	10
External test (GSE98918)	APM	0.419	0.244	12
External test (GSE98918)	OA	0.386	0.180	12
Mechanotransduction (GSE165874)	Control	0.520	0.082	9
Mechanotransduction (GSE165874)	Strain	0.604	0.071	9
Stability (GSE129147)	Damaged	0.633	0.266	10
Stability (GSE129147)	Healthy	0.342	0.311	9

CDI, cartilage degradation index.

When examining the CDI values summarized in Table 8, a clear divergence is observed between the low CDI scores (0.03–0.09) of healthy samples (Healthy, Normal, Preserved) in the training cohort and the high CDI score (0.93) of advanced-stage OA samples. In the external validation cohorts, APM samples were positioned in the intermediate-risk region as expected. The marginally higher mean CDI value of the APM group (0.419) compared to the OA group (0.386) in the GSE98918 cohort is due to the relatively early clinical features of OA samples in this specific cohort and the fact that the difference between the two groups did not reach a statistically significant level. The mean CDI value of tissues exposed to mechanical stress (Strain) (0.603) is higher compared to the control group (0.520). Similarly, the mean CDI value of damaged tissues (0.632) was higher compared to healthy tissues (0.341). These findings demonstrate that the model can detect not only advanced OA but also biomechanical deterioration due to mechanical loading and early tissue damage.

### 3.3 SHAP-based feature importance and mechanosensitivity

**FIGURE 4:**
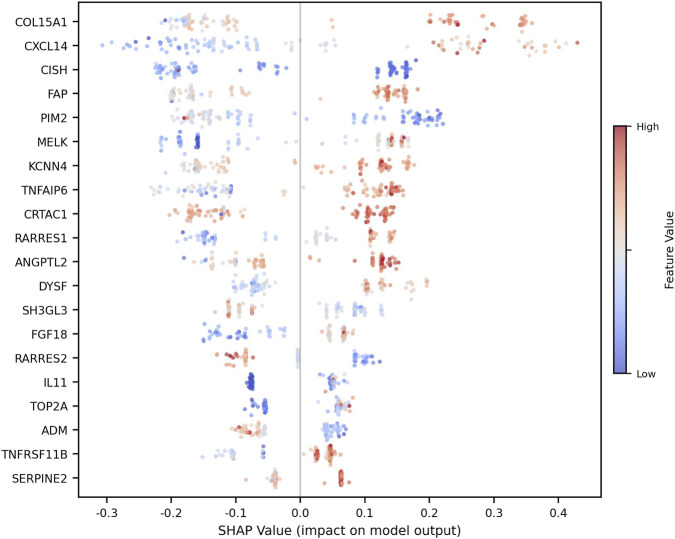
SHAP beeswarm plot showing the contribution of the top 20 genes to ensemble model predictions.

To clarify the decision-making mechanism of the developed ensemble model and to identify the key biological factors driving cartilage degradation, SHAP analysis was performed. This analysis quantitatively reveals the individual contribution (direction and magnitude of effect) of each gene to the model prediction. The distribution of the top 20 genes with the highest effects, according to the SHAP values obtained from the training set, is visualized in Figure 4.

**FIGURE 5:**
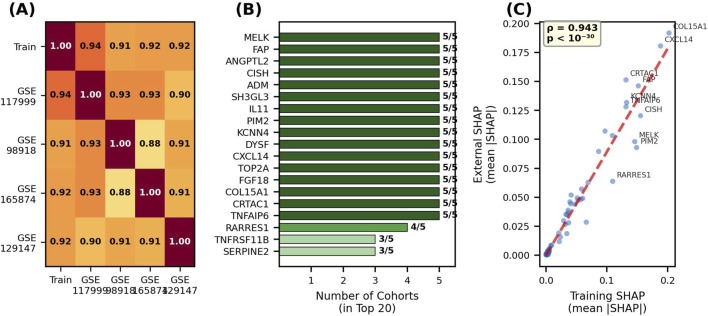
Cross-cohort consistency of SHAP-based feature importance. **(A)** Spearman correlation heatmap between cohorts. **(B)** Number of cohorts in which each gene appears in the top 20. **(C)** Scatter plot of training vs. independent validation cohorts mean SHAP values.

As shown in the beeswarm plot presented in Figure 4, the genes contributing most to the predictive power of the model were identified as COL15A1, CXCL14, CISH, FAP, and PIM2, respectively. Examining the color coding in the graph (red: high expression; blue: low expression), it can be seen that high expression levels of the COL15A1, CXCL14, and FAP genes produce positive SHAP values, directly leading the model toward the “degradation/OA” category. In contrast, low expression levels of the CISH and PIM2 genes were associated with a risk of degradation. To confirm the stability of these biomarkers identified by the model across different patient populations, SHAP significance scores were compared between the training set and independent validation cohorts, and the results are summarized in Figure 5.

As shown in Figure 5, the gene importance ranking learned by the model exhibits remarkably high consistency across different cohorts. The correlation heatmap in Figure 5A shows that the Spearman correlation coefficients (ρ) between the training set and independent validation cohorts range from 0.91 to 0.94. The gene consistency analysis presented in Figure 5B reveals that 16 of the top 20 most important genes appear in the “Top 20″list across all five cohorts (5/5). The scatter plot in Figure 5C confirms the strong linear relationship (ρ = 0.943, p < 10^−30^) between the mean SHAP values of the training set and the independent validation cohorts. These findings demonstrate that the model captures the fundamental biological mechanisms of cartilage degradation rather than memorizing dataset-specific artifacts (overfitting). To evaluate the mechanosensitivity of the selected 93 genes to mechanical stress, *Mechanosensitivity Scores* calculated from the *in vitro* mechanotransduction cohort (GSE165874) and the tissue stability cohort (GSE129147) are presented in Tables 9 and 10.

**TABLE 9:** SHAP top 20 genes.

Gene	Consensus SHAP	Rank	Log2FC	FDR
COL15A1	0.202	1	0.527	3.46e-10
CXCL14	0.189	2	0.709	3.01e-07
CISH	0.156	3	−0.606	4.40e-05
FAP	0.152	4	0.628	4.37e-08
PIM2	0.149	5	−0.515	1.85e-05
MELK	0.147	6	0.591	2.54e-05
KCNN4	0.133	7	0.526	1.83e-09
CRTAC1	0.132	8	0.565	3.35e-07
TNFAIP6	0.132	9	0.770	5.63e-11
RARRES1	0.110	10	0.640	7.84e-06
ANGPTL2	0.110	11	0.529	2.18e-05
DYSF	0.097	12	0.669	4.86e-09
SH3GL3	0.087	13	−0.597	3.09e-06
FGF18	0.069	14	0.530	6.54e-06
RARRES2	0.067	15	−0.719	9.05e-06
IL11	0.061	16	1.402	8.23e-07
TOP2A	0.059	17	0.843	8.33e-07
ADM	0.058	18	−0.693	6.58e-08
TNFRSF11B	0.053	19	0.761	4.02e-06
SERPINE2	0.052	20	0.646	3.47e-07

**TABLE 10:** Mechanosensitivity summary.

Category	N Genes	Mean mechano score	Mean SHAP	Top 5 genes
High mechanosensitivity	7	0.648	0.158	CXCL14, COL15A1, CISH, PIM2, MELK
Moderate mechanosensitivity	9	0.382	0.098	KCNN4, CRTAC1, TNFAIP6, ANGPTL2, DYSF
Low mechanosensitivity	77	0.046	0.012	MXRA5, IL11, SERPINE2, TOP2A, NGF

When examining the SHAP values of the 20 most important genes listed in Table 9, it can be seen that the COL15A1 (0.202) and CXCL14 (0.189) genes have a dominant role in model decisions. According to the mechanosensitivity classification summarized in Table 10, 7 of the 93 genes were in the *High Mechanosensitivity* category (e.g., CXCL14, COL15A1, CISH), nine were in the *Moderate Mechanosensitivity* category (e.g., KCNN4, CRTAC1), and 77 were in the *Low Mechanosensitivity* category. The fact that the CXCL14 and COL15A1 genes ranked highest in both the overall SHAP importance ranking and mechanosensitivity scores indicates that genetic responses to mechanical stress play a critical role in the early stages of biomechanical degradation in cartilage tissue.

### 3.4 Pathway enrichment and SHAP-weighted scoring

**FIGURE 6:**
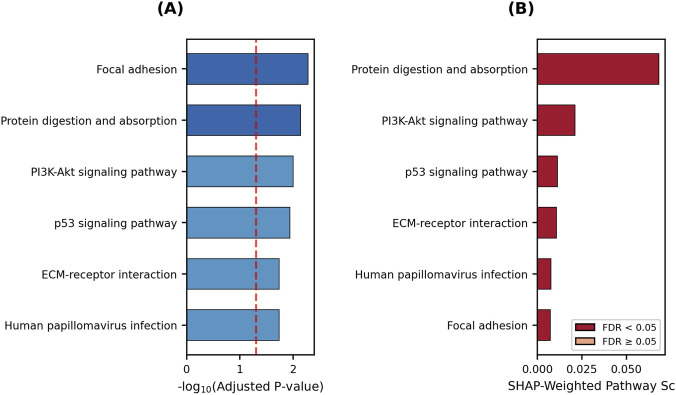
KEGG pathway enrichment analysis. **(A)** Enriched pathways for all 93 DEGs ranked by statistical significance. **(B)** SHAP-weighted pathway scores reflecting model-driven biological relevance.

To understand the biological mechanisms driving cartilage degradation at the system level, pathway enrichment analysis was performed on the 93 selected genes. In addition to the standard enrichment analysis, SHAP-weighted pathway scoring was applied to reflect the importance of each gene in the model. The results of these analyses are visualized in Figure 6.

As shown in the standard KEGG pathway analysis presented in Figure 6A, the pathways showing the strongest association with cartilage degradation were identified as “Focal adhesion” (FDR = 0.005) and *Protein digestion and absorption* (FDR = 0.007). Additionally, the *PI3K–Akt signaling pathway* (FDR = 0.010) and *p53 signaling pathway* (FDR = 0.011), which play a critical role in cell survival and stress response, were also significantly enriched. The SHAP-weighted scoring in Figure 6B shows which pathways cluster the genes that contributed most to the model’s predictive power. According to this analysis, the *Protein digestion and absorption* pathway stood out in both the standard analysis and the SHAP-weighted evaluation, confirming its central role in cartilage matrix degradation. To examine the model’s decision-making mechanism more deeply within a biological context, the SHAP-weighted pathway scores directly related to cartilage biology are summarized in Table 11.

**TABLE 11:** SHAP-weighted pathway scores (selected biologically relevant pathways).

Term	SHAP score	Adjusted P-value
JAK-STAT signaling pathway	0.156	0.268
Prolactin signaling pathway	0.156	0.258
Acute myeloid leukemia	0.149	0.258
Pathways in cancer	0.149	0.400
Cytokine-cytokine receptor interaction	0.088	0.040
Endocytosis	0.087	0.268
STAT3 pathway	0.079	0.040
Protein digestion and absorption	0.068	0.005
Collagen chain trimerization	0.068	0.024
Syndecan 1 pathway	0.068	0.021

As shown in Table 11, the SHAP-weighted analysis revealed that inflammatory response pathways such as the *JAK–STAT signaling pathway* (SHAP score = 0.156) and *Cytokine–cytokine receptor interaction* (SHAP score = 0.087) achieved the highest impact values. This indicates that the machine learning model uses not only structural matrix degradation but also early-stage inflammatory signals (particularly via CXCL14 and IL11) that trigger this degradation as primary decision factors when predicting cartilage degradation.

## 4 Discussion

In this study, an ensemble machine learning model supported by explainable artificial intelligence (SHAP), capable of predicting the transcriptomic signatures of biomechanical degradation from cartilage tissue, was developed. As the primary original contributions of this work, two novel analytical metrics were introduced: the Mechanosensitivity Score, which quantitatively ranks the sensitivity of individual genes to mechanical stress based on SHAP values, and the SHAP-weighted pathway score, which prioritizes biological mechanisms that actively drive the model’s predictive performance. It should be emphasised that the Cartilage Degradation Index (CDI) serves as a molecular proxy reflecting the cellular response to mechanical stress, rather than a direct measurement of physical mechanical properties. While existing studies in the literature generally treat osteoarthritis (OA) as a binary classification problem (sick vs. healthy), this study defined a continuous CDI reflecting the progressive nature of the disease. The developed model showed high discrimination (AUC = 0.960) in 5-fold cross-validation, and its performance was validated in four independent validation cohorts. In particular, the significant increase in CDI score in chondrocytes subjected to *in vitro* mechanical stress and in early-stage damaged tissues demonstrates that the model can detect not only advanced structural destruction but also early biomechanical deterioration. The mechanosensitive gene profile (COL15A1, CXCL14, CISH, FAP, PIM2) determined by SHAP analysis and SHAP-weighted pathway scoring offers a new perspective on the molecular mechanisms underlying cartilage degradation. These findings represent a significant step toward monitoring cartilage health at the molecular level and developing digital biomarkers for next-generation smart orthopedic devices.

The analysis of transcriptomic data using machine learning has gained significant momentum in OA research in recent years. For example, Zhao et al. (2024) developed a diagnostic model based on four genes (CRTAC1, DIO2, ANGPTL2, MAGED1) in cartilage tissue, while Zhao J et al. (2026) identified multiple tissue biomarkers using SHAP analysis. Similarly, He et al. (2025) utilized various machine learning algorithms to identify immune–metabolic gene signatures. However, these studies primarily focused on classifying the disease into two extremes: “healthy” and “osteoarthritic.” Yet, cartilage degradation is a continuous biomechanical process triggered by mechanical stress and progressing over time. The CDI score presented in this study aims to reflect this process by modeling degradation as a continuous metric between 0 and 1. In the external validation cohorts, the placement of APM patients representing early-stage damage, and cells exposed to mechanical stress, in the intermediate-risk zone between healthy and advanced OA supports the compatibility of this continuous modeling approach with biological progression.

SHAP analysis, used to clarify the decision-making mechanism of the model, highlighted critical mechanosensitive genes (COL15A1, CXCL14, CISH, FAP, PIM2) involved in cartilage degradation. The literature reports that COL15A1 is upregulated in osteoarthritic cartilage and plays a role in ECM remodeling (Karlsson et al., 2010). Similarly, FAP (Fibroblast Activation Protein) expression has been shown to increase in chondrocytes after pro-inflammatory stimuli and to accelerate cartilage degradation (Milner et al., 2006; Fan et al., 2023). On the other hand, the association of low expression levels of the CISH gene, which suppresses cytokine signaling, and the PIM2 kinase, which regulates apoptosis, with degradation risk provides important clues about how mechanical stress disrupts the anabolic–catabolic balance (Meng et al., 2022). The SHAP-weighted pathway scoring presented in this study goes beyond standard enrichment analyses, confirming that the key biological processes driving the model’s predictions are inflammatory and catabolic mechanisms such as *protein digestion and absorption* and the *JAK–STAT signaling pathway*.

The CDI and the mechanosensitive gene profile described in this study provide a conceptual framework for the future development of next-generation smart orthopedic devices in the field of translational biomechanics. Currently, research on sensor-integrated smart implants capable of measuring intra-articular loading and contact pressure is rapidly increasing (Iyengar et al., 2021; Nash et al., 2022). However, these devices generally monitor only physical parameters (force, pressure, temperature) and cannot assess the tissue’s biological response. The developed machine learning model converts the molecular-level response to mechanical stress (particularly through genes such as COL15A1 and CXCL14) into a quantitative score (CDI), theoretically bridging the gap between physical sensor data and biological tissue degradation. It is crucial to acknowledge that the direct *in vivo* measurement of transcriptomic profiles (i.e., real-time gene expression sequencing) within a joint is currently not feasible in routine clinical practice. Therefore, translating these computational findings into smart monitoring devices requires a multi-step technological roadmap. The first essential step is the validation of these transcriptomic signatures at the protein or metabolite level in minimally invasive liquid biopsies, such as synovial fluid or peripheral blood (Budd et al., 2018; Mobasheri et al., 2023). Once the corresponding protein biomarkers for genes like COL15A1 and CXCL14 are validated, the second step involves the development of point-of-care (POC) microfluidic biosensors capable of detecting these specific targets (Zou et al., 2024). Finally, recent advancements in implantable biochemical sensors, such as interface-adapted fiber sensors that can continuously monitor synovial fluid components *in vivo* (Li et al., 2026), offer a promising technological avenue. By integrating the CDI algorithm with such emerging implantable biochemical sensors, future smart orthopedic devices could theoretically monitor both physical loads and the resulting biological degradation in real-time, enabling personalized rehabilitation protocols that dynamically adapt to patient-specific biomechanical limits.

One of the study’s most important methodological strengths is that the model learns directly from biomechanical degradation signals rather than inter-patient genetic variations by using damaged and preserved tissues from the same patients in the training cohort. Furthermore, while the overall sample size (n = 203) is relatively modest for high-dimensional transcriptomic modeling, rigorous methodological steps were taken to ensure model robustness and prevent overfitting. These included aggressive dimensionality reduction from 10,283 genes to 93 biologically significant DEGs, 5-fold stratified cross-validation, and most importantly, successful validation across four completely independent external cohorts—including different geographic populations and *in vitro* mechanical stress models—that were never exposed to the training phase. In addition, the permutation test (p < 0.001) and bootstrap analysis (Ensemble AUC 95% CI: 0.900–0.996) provided strong statistical evidence that the model’s predictive power is highly stable and not driven by chance. However, the study also has some limitations. First, the analyses are based on retrospective transcriptomic data obtained from publicly available databases (GEO); therefore, the predictive value of the CDI score in clinical practice needs to be validated in prospective cohort studies. Second, since the data used reflect tissue-level (bulk) gene expression, cellular heterogeneity at single-cell resolution could not be fully assessed. Future studies examining the levels of the identified mechanosensitive genes (COL15A1, CXCL14, etc.) in minimally invasive samples such as joint fluid or blood (liquid biopsy) will improve the clinical applicability of the model. Furthermore, elucidating the functional roles of these genes in *in vivo* mechanistic loading models and integrating the developed machine learning algorithms into wearable biosensors or smart implant systems will open new horizons for the early diagnosis and personalized treatment of osteoarthritis.

## 5 Conclusion

This study presents an innovative machine learning approach, supported by explainable artificial intelligence, that models the molecular response to cartilage degradation as a continuous process. Cartilage Degradation Index can detect early-stage molecular changes and cellular responses to mechanical stress with high accuracy. The identified mechanosensitive gene profile (COL15A1, CXCL14, CISH, FAP, PIM2) provides novel mechanistic insights into the disruption of the anabolic–catabolic balance in osteoarthritis pathogenesis. These findings provide an important foundation for monitoring cartilage health at the molecular level, developing early diagnostic strategies, and creating digital biomarkers for next-generation smart orthopedic implants.

## Data Availability

Publicly available datasets were analyzed in this study. This data can be found here: The datasets analyzed in this study are publicly available in the Gene Expression Omnibus (GEO) database under the accession numbers GSE57218, GSE114007, GSE169077, GSE117999, GSE98918, GSE165874, and GSE129147. The complete analysis pipeline, processed data, and source code supporting the findings of this study are available in the Zenodo repository: https://doi.org/10.5281/zenodo.19009050.
